# Temperature Fluctuations Modulate Molecular Mechanisms in Skeletal Muscle and Influence Growth Potential in Beef Steers

**DOI:** 10.1093/jas/skad343

**Published:** 2023-10-04

**Authors:** Zachary K Smith, Erika Eckhardt, Won Seob Kim, Ana Clara Baio Menezes, Warren C Rusche, Jongkyoo Kim

**Affiliations:** Department of Animal Science, South Dakota State University, Brookings, SD, USA; Department of Animal Science, Michigan State University, East Lansing, MI, USA; Department of Animal Science, Michigan State University, East Lansing, MI, USA; Department of Animal Science, South Dakota State University, Brookings, SD, USA; Department of Animal Science, South Dakota State University, Brookings, SD, USA; Animal Science and Food Science and Human Nutrition, Michigan State University, East Lansing, MI, USA

**Keywords:** thermal stress, beef cattle, skeletal muscle, adipose tissue

## Abstract

Our investigation elucidated the effects of severe temperature fluctuations on cellular and physiological responses in beef cattle. Eighteen Red Angus beef steers with an average body weight of 351 ± 24.5 kg were divided into three treatment groups: 1) Control (CON), exposed to a temperature-humidity index (THI) of 42 for 6 h without any temperature changes; 2) Transport (TP), subjected to a one-mile trailer trip with a THI of 42 for 6 h; and 3) Temperature swing (TS), exposed to a one-mile trailer trip with a THI shift from 42 to 72–75 for 3 h. Our findings indicate that TS can induce thermal stress in cattle, regardless of whether the overall temperature level is excessively high or not. Behavioral indications of extreme heat stress in the cattle were observed, including extended tongue protrusion, reduced appetite, excessive salivation, and increased respiratory rate. Furthermore, we observed a pronounced overexpression (*P* < 0.05) of heat shock proteins (HSPs) 20, 27, and 90 in response to the TS treatment in the longissimus muscle (LM). Alterations in signaling pathways associated with skeletal muscle growth were noted, including the upregulation (*P* < 0.01) of Pax7, Myf5, and myosin heavy chain (MHC) isoforms. In addition, an increase (*P* < 0.05) in transcription factors associated with adipogenesis was detected (*P* < 0.05), such as PPARγ, C/EBPα, FAS, and SCD in the TS group, suggesting the potential for adipose tissue accumulation due to temperature fluctuations. Our data illustrated the potential impacts of these temperature fluctuations on the growth of skeletal muscle and adipose tissue in beef cattle.

## Teaser

Our study demonstrated how temperature swings can induce thermal stress in beef cattle, leading to observable behavioral and physiological changes. Our findings revealed the impact of severe temperature fluctuations on the signaling pathways involved in skeletal muscle growth and the potential for adipose tissue accumulation. While gaining insight into the cellular and physiological responses of beef cattle to temperature variations, elucidated on the complex interplay between environmental factors and animal productivity.

## Introduction

Thermal stress refers to temperature fluctuations that, within certain limits, can have adverse or fatal effects on animals. Escalating temperatures pose economic challenges for cattle producers, leading to losses such as decreased reproduction, reduced feed intake, and poor growth performance indicators. These losses can ultimately result in increased veterinary costs, reduced weight gain, performance productivity, animal welfare concerns, higher mortality rates, and meat quality defects. It is estimated that heat stress (HS)-related economic losses could reach $39.94 billion per year by the end of the 21st century (St-Pierre et al., 2003). The beef cattle industry alone suffers annual losses of around $370 million due to animal mortality caused by (Thornton et al., 2022). Humidity and temperature significantly impact HS, and the temperature-humidity index (THI) is used to measure its effects. A THI greater than 72 indicates mild stress, while a THI above 89 results in severe stress (Armstrong, 1994; Kim et al., 2021). Cattle maintain a constant body temperature within a thermoneutral zone (TNZ) of ambient temperatures. For instance, yearling *Bos taurus* cattle fed on an energy-dense diet have a TNZ of −10 °C to 20 °C. Any temperature above or below this range induces stress on the animal, requiring additional energy expenditure to maintain homeostasis (Vaughn, 2012; Kim et al., 2022).

Producers are experiencing significant losses because of the intensification of climate change, particularly in regions where sudden and extreme temperature fluctuations occur, affecting animals that are not accustomed to thermal stress or genetically predisposed to it. In recent years, the United States has experienced abrupt temperature changes, moving from cold to warm and returning to cold. Feedlot cattle deaths as a result of temperature stress for Iowa, Kansas, and Nebraska are a continued threat with global temperature swings occurring with severe impacts to producers being felt. The summer of 2011 saw approximately 4,000 feedlot cattle mortalities as a result of heat indexes climbing over 43 °C (FarmProgress, 2011). More recently, June of 2022 saw heat waves in Kansas lead to over 2,000 dying as a result of excess temperatures and humidity (Polansek, 2022).

There is a lack of sufficient research exploring the effects of temperature fluctuations on postnatal muscle growth and adipose tissue metabolism in bovine species. Previous studies have primarily focused on immune responses and metabolic disorders in dairy cattle resulting from HS. However, it is crucial to understand these effects to improve meat quality and yield in the beef industry. We hypothesized that extreme temperature swings might modify the characteristics of skeletal muscle and adipose tissue by influencing cellular signaling pathways. Therefore, this study aimed to investigate the impact of acute temperature changes on attributes related to skeletal muscle and adipose tissue, as well as the physiological responses of beef cattle.

## Material and methods

### Animal care and diet information

The South Dakota State University Institutional Animal Care and Use Committee (IACUC; protocol number [2312-012A]) approved all animal procedures and sampling protocols for this study.

A total of 18 Red Angus beef steers (mean body weight: 351 ± 24.5 kg) were selected from the South Dakota State University Ruminant Nutrition Center for an acute induction HS study. The induction of HS was carried out at the South Dakota State University Animal Wing (AW). The steers were individually weighed and randomly assigned to one of three treatment groups: 1) Control (CON): This group consisted of steers that remained at the SDSU feedlot and were exposed to a THI of 42 for a duration of 6 h. The average temperature during this period was 3.6 °C with 65% relative humidity (RH). 2) Transport (TP): This group served as the negative control. The steers were transported one mile from the feedlot to the AW (indoor) and then returned to the feedlot without temperature changes. They were exposed to a THI of 42 for 6 h, with an average temperature of 3.6 °C and 65% RH. 3) Temperature swing (TS): The steers in this group experienced environmental changes. After the one-mile trailer trip from the feedlot, done simultaneously with the TP group steers to AW, the THI shifted from 42 to 72–75. They were exposed to an average temperature of 26.8 °C with 58% RH for 3 h. Steers in the TS group were allocated to an individual pen with sufficient space, provided with TMR (8 kg); however, feed was untouched and no measurements were recorded, and the room was equipped to adjust temperatures to reach the desired THI and humidity levels.

THI was calculated based on Hahn et al. (2003) using the following formula,


$$\begin{array}{l} \begin{array}{lll} & THI\;=\;0.8\;\times \;ambient\;temperature\;+\; & \;\left[\left(\%\;relative\;humidity/100\right)\;\times \;\left(ambient\;temperature\;-14.4\right)\right]\;+46.4.\; \end{array} \end{array}$$

Steers were fed a diet (dry matter (DM) basis) based upon high-moisture ear corn (HMC; 70%), dried distillers grains plus solubles (DDGS; 15%), hay (10%), and a molasses-based suspended supplement (5%). The liquid supplement provided vitamins and minerals to meet or exceed all nutrient requirements for growing-finishing steers, according to the National Academies of Sciences and Medicine (2016) and also provided monensin sodium 33.08 mg/kg of diet DM. Fresh feed was manufactured twice daily in a stationary horizontal mixer (2.35 m^3^; Roto-Mix, Dodge City, KS; scale readability ± 0.45 kg) and offered to steers in a 50:50 split at 0800 h and 1400 h. Individual ingredient samples were collected weekly, and DM was calculated following drying in a 60 °C forced air oven until no weight change to calculate dry matter intake (DMI). Proximate analysis of ingredients was conducted using samples collected the week of the experiment: DM [method no. 935.29 (AOAC, 2000)], *N* [method no. 968.06 (AOAC, 2016); Rapid Max N Exceed, Elementar, Mt. Laurel, NJ], and ash [method no. 942.05 (AOAC, 2000)]. Ether extract (EE) content analysis was conducted for DDGS utilizing an Ankom Fat Extractor (XT10; Ankom Technology, Macedon, NY); tabular values were used for the remainder of the ingredients (National Academies of Sciences and Medicine, 2016). Acid detergent fiber (ADF) and neutral detergent fiber (NDF) percentages were estimated to be 3% and 9%, respectively, for corn; fiber content analysis for all other ingredients was conducted as described by Goering and Van Soest (1970). The diet provided 11.8% CP, 18.9% NDF, 9.6% ADF, 4.9% ash, 4.0% EE, 1.97 Mcal/kg of net energy for maintenance (NEm), and 1.33 Mcal/kg of net energy for gain (NEg).

### Blood sampling for sera harvest and cortisol determination

Whole blood was collected via the jugular vein at 0 and 3 h relative to HS treatment. Venipuncture blood was collected using an 18-gauge × 1.5 in. (VACUETTE, Greiner Bio-One North America Inc., Monroe, North Carolina, USA) into evacuated BD Vacutainer Serum Tubes (Becton, Dickson, and Company, Franklin Lakes, NJ, USA) for serum separation. Blood was allowed to clot for 24 h at 4 °C and then centrifuged for 20 min (1250 × *g* at 4 °C) for sera separation. The sera were used to quantify circulating concentrations of cortisol via radioimmunoassay procedures. The inter-and intra-assay CV was less than 10% for each, respectively. Samples were considered for re-runs if the CV between triplicate determinations was greater than 10%.

### Rectal temperature and respiratory rate measurement

Rectal temperature was determined by a trained technician immediately prior to collection of the skeletal muscle biopsy. To track HS, respiratory rate (RR) was evaluated via manual observation initially and at h 1, 2, and 3 for TS steers. This was done using a stopwatch recording the time required for 10 full flank movements. RR as beat per minute (bpm) was calculated as 600/time in seconds required for 10 full flank movements.

### Muscle biopsies

Biopsy samples were collected from the longissimus muscle (LM) of steers at 0 h (*n* = 6; 2 steers/treatment) and 3 h (*n* = 18; 6 steers/treatment) relative to HS treatment. Steers undergoing the first biopsy (0 h) were collected from the steers’ right side, while the second biopsy (3 h) was collected from the steers’ left side. All biopsy samples were collected between the 12th and 13th rib using a custom-made biopsy needle (6 mm Bergstrom) following the modified procedures outlined in Smith et al. (2019). Samples immediately after harvest were placed in cryovials, snap-frozen, and stored in a Whirl-Pak (Nasco, Fort Atkinson, WI) on dry ice until transport to Smith Lab, South Dakota State University. Samples were packed on dry ice and shipped to the Kim Lab, Michigan State University (MSU), for analysis. Upon reception at the Kim Lab, MSU, approximately 0.1 g of tissue was aliquoted into snap-top tubes and stored at −80 °C until further analysis.

### mRNA isolation and RT-qPCR

Biopsied LM tissue samples used for mRNA quantification were thawed and extracted via TRIzol reagent (Invitrogen, Carlsbad, CA, USA) following a protocol modified by Smith et al. (2019). After extraction, mRNA purity and concentration were determined using a NanoDrop One/One Microvolume UV-Vis Spectrophotometer (Thermo Fisher Scientific) with absorbance set at 260 nm/280 nm. The 260 nm/280 nm ratio was established at an acceptable range of 2.00 to 2.23. The QuantiTect reverse transcription kit (Qiagen, Germantown, MD, USA) was used for Genomic DNA removal and cDNA synthesis in collaboration with manufacturers’ guidelines. Relative quantification (RQ), value of cDNA was evaluated using TaqMan Fast Advanced Master Mix (Applied Biosystems) and TaqMan Gene expression Assays (Thermo Fisher Scientific) (Table 1). Real-time quantitative PCR (RT-qPCR) (QuantStudio 6Pro, Applied Biosystems) was used to evaluate gene expressions of *heat shock proteins (HSP)*, *20*, *27*, *70*, and *90*, *myogenic factor 5 (Myf5)*, *IGF-1*, *ribosomal protein S6 kinase B1 (P70*^*S6K*^), *myogenic differentiation (MyoD)*, *paired box 7 (Pax7)*, *myogenin (MyoG)*, *CCAAT/enhancer-binding protein alpha* (*C/EBPɑ)*, *fatty acid synthetase (FAS)*, *peroxisome proliferator-activated receptor gamma (PPARγ)*, *stearoyl-coenzyme A desaturase (SCD)*, and *myosin heavy chains (MHC) I*, *IIA*, and *IIX*. Housekeeping genes of *ribosomal protein subunit 9* (*RPS9*) and *hydroxymethylbilane synthase* (*HMBS*) were used to quantify relative expression against the quantity of genes of interest within the samples as endogenous controls. *RPS9* and *HMBS* are considered acceptable for use as housekeeping genes due to the lack of diversity of *RPS9* and *HMBS* across skeletal muscle tissue in cattle (Kim et al., 2018). cDNA relative quantification was evaluated in triplicate using recommended thermal cycler parameters at 45 cycles of 15 s at 95 °C and 1 min at 60 °C. All analyses of real-time results and Ct values were analyzed using QuantStudio 6 Pro system (Applied Biosystems).

**Table 1.** TaqMan probes and primers used for RT-qPCR assays

Gene^1^	TaqMan probe assay	Manufacturer
*RPS9*	Bt03272016_m1	Thermo Fisher
*HMBS*	Bt03234763_m1	
*HSP20*	Bt03213719_m1	
*HSP27*	Bt03220563_m1	
*HSP70*	Bt03292670_g1	
*HSP90*	Bt03244099_g1	
*MyoD*	Bt03244740_m1	
*MyoG*	Bt03258928_m1	
*IGF-1*	Bt03252282_m1	
*Myf5*	Bt03223134_m1	
*P70* ^*S6K*^	Bt00923436_m1	
*PPARγ*	Bt03217547_m1	
*C/EBPα*	Bt03224529_s1	
*FAS*	Bt03210481_m1	
*SCD*	Bt04307476_m1	
*MHC Ⅰ*	Bt03224257_m1	
Genes	Primer and probe sequence (5’ to 3’)	Manufacturer
*Pax7*		
Forward	GCCCTCAGTGAGTTCGATTAG	
Reverse	GATGCTGTGCTTGGCTTTC	
Probe	6FAM-TTCGTCCTCCTCCTCCTTCTTCCC-MGBNFQ	
*MHC ⅡA*		Thermo Fisher
Forward	GCAATGTGGAAACGATCTCTAAAGC	
Reverse	GCTGCTGCTCCTCCTCCTG	
Probe	6FAM-TCTGGAGGACCAAGTGAACGAGCTGA-TAMRA	
*MHC ⅡX*		
Forward	GGCCCACTTCTCCCTCATTC	
Reverse	CCGACCACCGTCTCATTCA	
Probe	6FAM-CGGGCACTGTGGACTACAACATTACT-TAMRA	

^1^ *RPS9*: ribosomal protein subunit 9; *HMBS*: hydroxymethylbilane synthase; *HSP*: heat shock protein; *MyoD*: myoblast determination protein 1; *MyoG*: myogenin; *IGF-1*: insulin-like growth factor 1; *Myf5*: myogenic factor 5; *P70*^*S6K*^: ribosomal protein S6 kinase beta 1; *PPARγ*: peroxisome proliferator activated receptor gamma; *C/EBPα*: CCAAT/enhancer-binding protein-alpha; *FAS*: fatty acid synthase; *SCD*: stearoyl-coenzyme A desaturase; *Pax7*: paired box gene 7; *MHC*: myosin heavy chain.

### Western blotting

Tissue samples previously stored in a −80 °F freezer were thawed and prepped using mammalian protein extraction lysate buffer (M-PER) (Thermo Fisher Scientific) in addition to protein inhibitor (Thermo Fisher Scientific). A NanoDrop One/OneC Microvolume UV–Vis Spectrophotometer (Thermo Fisher Scientific) was used for quantification of total protein via bicinchoninic acid assay (BCA, Thermo Fisher Scientific), 562 nm was set as the resonance level. For analysis, 20 µg of protein sample was denatured for 10 min at 70 °C and 2 min at 85 °C prior to loading onto Bolt 4%–12% Bis-Tris Plus gel (Thermo Fisher Scientific). Gels ran at 200 V for 22 min before being transferred onto a nitrocellulose membrane using iBlot 2 Dry Blotting System (Thermo Fisher Scientific). Using the iBind Flex Solution (iBind Flex Buffer, Thermo Fisher Scientific), membranes were incubated at room temperature for 10 min prior to transfer to block nonspecific bindings. For this study, primary antibodies: anti-HSP70, mouse monoclonal, dilution of 1:1000 (Abcam Inc., Cambridge, MA, USA), anti-HSP27, mouse monoclonal, dilution of 1:1000 (Developmental ­Studies Hybridoma Bank; DSHB, Iowa City, IA, USA), anti-HSP20, mouse monoclonal, dilution of 1:1000 (DSHB), anti-HSP90, mouse monoclonal, dilution of 1:1000 (DSHB), anti-Pax7, mouse monoclonal, dilution of 1:1000 (DSHB), anti-Myf5, rabbit polyclonal, 1:1000 (Abcam Inc.), anti-IGF-1, rabbit polyclonal, dilution of 1:1000 (Bioss Inc., Boston, MA, USA), anti-PPARγ, rabbit polyclonal, dilution of 1:1000 (Cell Signaling, Danvers, MA, USA), anti-C/EBPα, rabbit polyclonal, dilution of 1:1000 (Cell Signaling), anti-SCD, rabbit polyclonal, dilution of 1:1000 (Cell Signaling), anti-FAS, rabbit polyclonal, 1:1000 (Cell Signaling), anti-mTOR, rabbit monoclonal, dilution of 1:1000 (Cell Signaling, Danvers, MA, USA), anti-phospho-mTOR, rabbit polyclonal, dilution of 1:1000 (Cell Signaling), anti-p70^S6K^, rabbit polyclonal, dilution of 1:1000 (Cell Signaling), anti-phospho-p70^S6K^, rabbit monoclonal, dilution of 1:1000 (Cell Signaling), anti-Akt, mouse monoclonal, dilution of 1:1000 (DSHB), anti-phospho-Akt, rabbit polyclonal, dilution of 1:1000 (Cell Signaling), and glyceraldehyde 3-phosphate dehydrogenase (GAPDH), mouse monoclonal, dilution of 1:1000 (DSHB). With secondary antibodies: Goat anti-mouse IgG H&L (HRP), mouse polyclonal, dilution of 1:1000 (Abcam Inc.) and Goat anti-rabbit IgG H&L (HRP), rabbit polyclonal, dilution of 1:2000 (Abcam Inc.). Using an iBind Flex Western System (Thermo Fisher Scientific), antibodies were applied at room temperature and incubated for 4 h. Upon which time, Western Blot bands were visualized using SuperSignal West Pico Chemiluminescent Substrate (Thermo Fisher Scientific). Fluorchem imager (Alpha Innotech, San Leandro, CA, USA) was used for signal detection. GAPDH or phospho-protein was used as a reference protein with associated protein expression of the proteins evaluated in question. Relative protein expression was quantified using ImageJ software (NIH, Bethesda, MD, USA).

### Statistical analysis

The completed randomized design (CRD) was employed to assign steers to the treatment group. The dependent variables were the skeletal muscle gene expression, protein levels, and blood parameters, behavior responses, and the independent variable was the treatment group.

The collected gene expression and protein level data were analyzed using one-way ANOVA. If a significant difference is found, the results of Tukey’s test will be reported, indicating which treatment groups differ significantly from each other using GraphPad Prism Version 9.5.0 (Graph Pad Software, San Diego, CA, USA). All results were reported using a least-squared means (LSMeans). Standard errors of means are used for data display, with an α value of 0.05 used for significance, with *P*-values between 0.05 and 0.10 denoted for tendencies.

## Results

### Behavioral changes and rectal temperature

**Figure 1.**
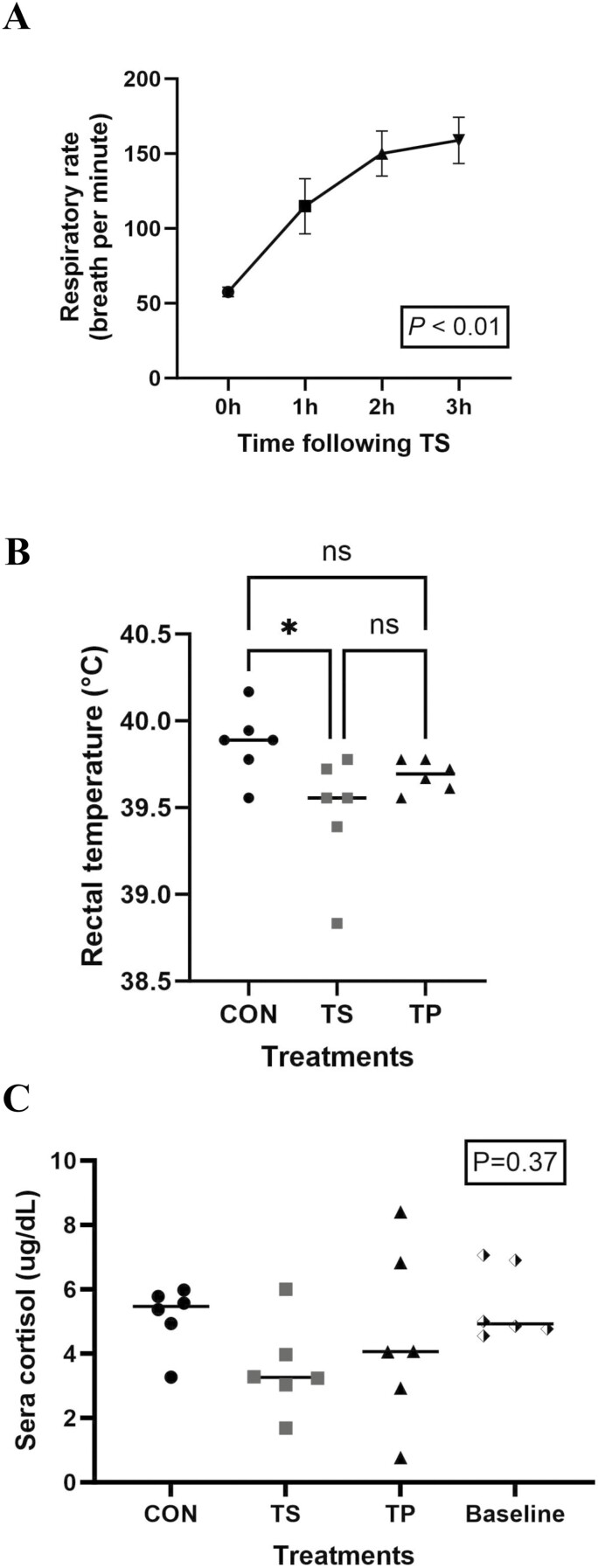
(A) Respiratory rate measured in cattle in the Temperature Swing (TS) group. (B) Rectal temperature measured 3 h after trial initiated. (C) Serum cortisol levels in steers: baseline levels measured in the morning before the trial started. Blood samples from the Control (CON), Temperature Swing (TS), and Transportation (TP) groups were taken after 3 h of treatment.

The steers in the TS group exhibited a significant (*P* < 0.01) increase in respiratory rate over time, ranging from 50 to 150 breaths per minute (Figure 1). After 2 h of exposure to TS conditions, the steers displayed tongue protrusion, and excessive salivation was observed. During this period, there was no food intake observed for any of the steers. Rectal temperatures were lowered (*P* < 0.05) in the TS group compared to the CON steer (Figure 1).

### Serum cortisol level

Neither sera cortisol level after 3 h of exposure to treatment nor the change from baseline was influenced by treatment (*P* ≥ 0.35) (Figure 1).

### Heat shock proteins

**Figure 2.**
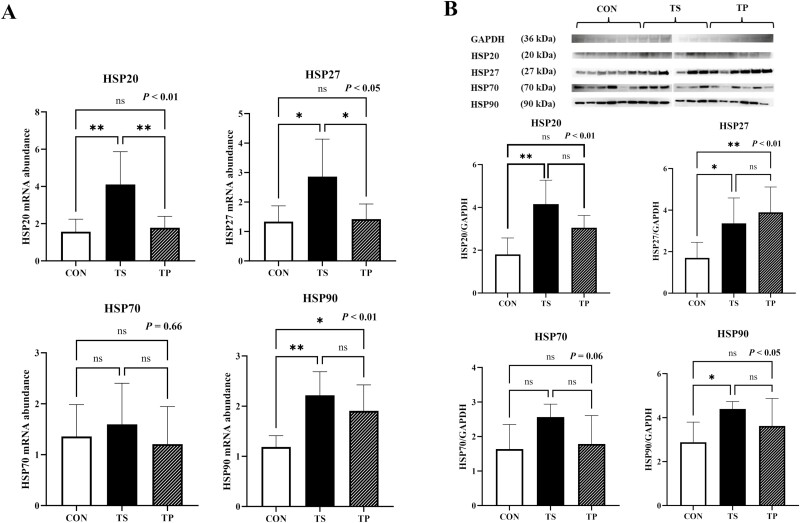
mRNA expression and protein abundance levels of heat shock protein (HSP) 20, 27, 70, and 90 in beef steers under temperature swing and transport conditions. (A) *HSP20*, *27*, *70,* and *90* mRNA levels in CON, TS, and TP groups. (B) Western blots (top) and quantification of protein levels (bottom) of HSP20, HSP27, HSP70, and HSP90 in CON, TS, and TP groups. Error bars indicate the standard error. The *P*-values are determined by one-way ANOVA (*P < 0.05, **P < 0.01).

In the TS group, steers exhibited a significant increase (*P* < 0.01) in the expression of *HSP20* (Figure 2A). HSP20 protein level was also increased (*P* < 0.01) in TS group compared to CON (Figure 2B). The expression of *HSP27* was also significantly increased in the TS group compared to both the CON and TP groups (*P* < 0.05) (Figure 2A). This finding was further supported by the protein analysis, which showed that HSP27 was increased (*P* < 0.01) in both the TS and TP groups compared to the CON group (Figure 2B). Steers in the TS and TP groups exhibited a significant increase (*P* < 0.01) in the gene expression of *HSP90* compared to the CON group (Figure 2A). HSP90 protein level was also increased (*P* < 0.05) in TS group compared to CON (Figure 2B). However, the mRNA expression of *HSP70* did not show any significant changes (*P* = 0.66) as a result of the treatment (Figure 2A). Nonetheless, there was a tendency (*P* = 0.06) for TS to increase the protein abundance of HSP70 in the TS group (Figure 2B).

### Myogenic regulatory factors (MRFs) and genes associated with muscle hypertrophy

**Figure 3.**
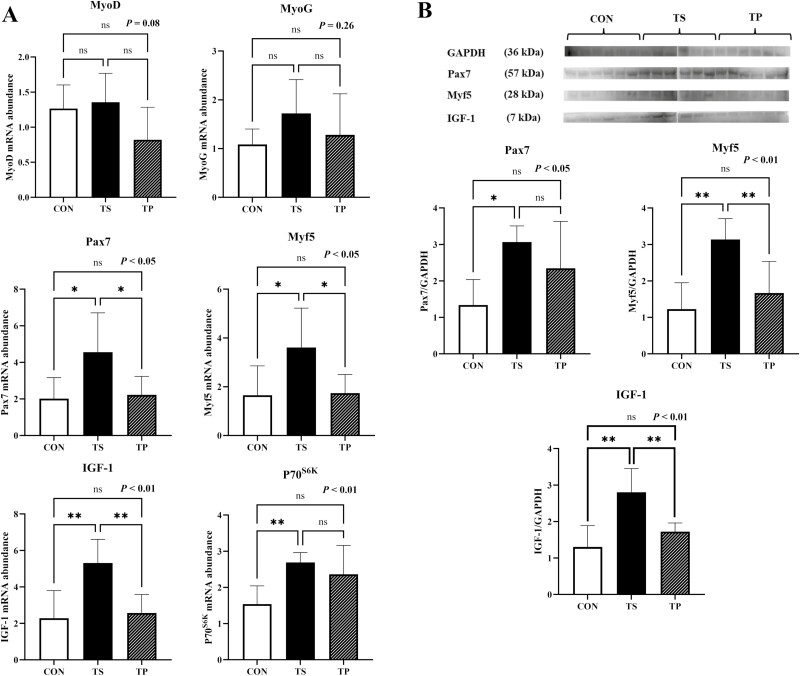
mRNA expression and protein abundance levels of myogenic regulatory factors (MRFs) (MyoD, MyoG, Myf5), Pax7, IGF-1, and P70^S6K^ in beef steers under temperature swing and transport conditions. (A) MRFs (MyoD, MyoG, Myf5), Pax7, IGF-1, and P70^S6K^ mRNA levels in CON, TS, and TP groups. (B) Western blots (top) and quantification of protein levels (bottom) of Pax7, Myf5, and IGF-1 in CON, TS, and TP groups. Error bars indicate the standard error. The *P*-values are determined by one-way ANOVA (*P < 0.05, **P < 0.01).

**Figure 4.**
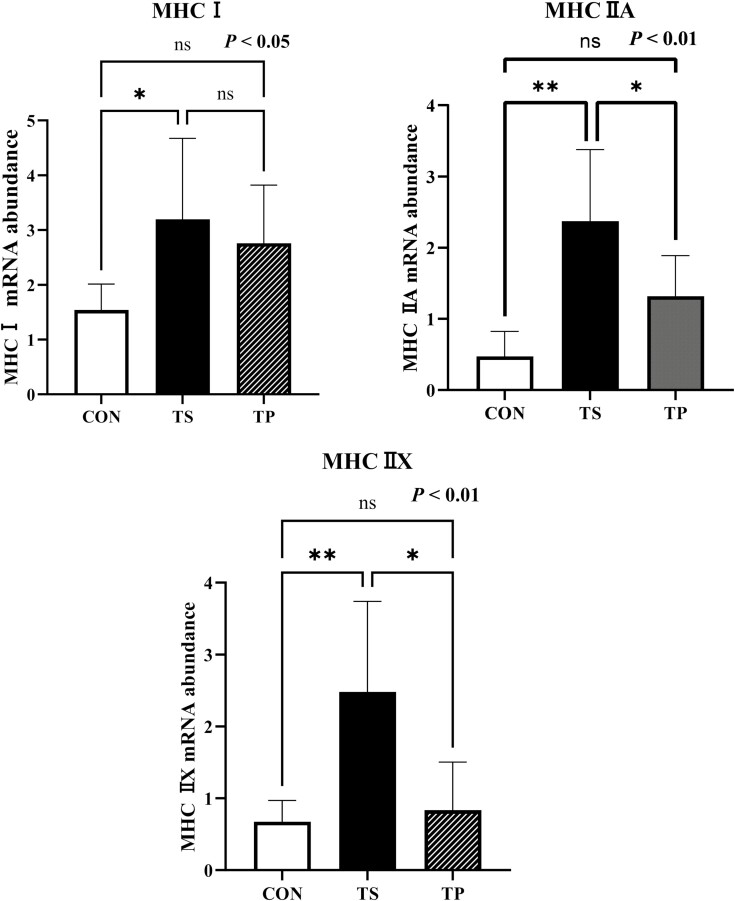
mRNA expression of myosin heavy chain (MHC) I, IIA, and IIX in beef steers under temperature swing and transport conditions. Error bars indicate the standard error. The *P*-values are determined by one-way ANOVA (*P < 0.05, **P < 0.01).

Under the TS condition, steers exhibited a significant upregulation (*P* < 0.05) in the mRNA gene expression of *Pax7* and *Myf5* (Figure 3A). This finding was further supported by the protein-level analysis, which demonstrated a significant increase (*P* < 0.05) in both Pax7 and Myf5 due to TS (Figure 3B). The expression of *MyoD* showed a tendency to decrease (*P* = 0.08) in the TP group. In contrast, no significant change was observed in *MyoG* levels (*P* = 0.26) (Figure 3A). Steers in the TS group demonstrated overexpression (*P* < 0.01) of *IGF-1* compared to the CON (Figure 3A). IGF-1 protein level was also increased (*P* < 0.01) in TS group compared to the CON and TP groups (Figure 3B). In addition, the expression of *P70*^*S6K*^ also increased significantly (*P* < 0.01) in the TS group compared to the CON group. However, it did not differ significantly from the TP group (Figure 3A). The TS treatment resulted in the upregulation of three isoforms of myosin heavy chains (MHCs) in LM (Figure 4). Specifically, the expression of *MHC I* was significantly increased (*P* < 0.05) in the TS group compared to the CON group. Furthermore, the TS group exhibited the highest expression (*P* < 0.01) of *MHC IIA* and the highest expression of *MHC IIX* among all the treatment groups.

**Figure 5.**
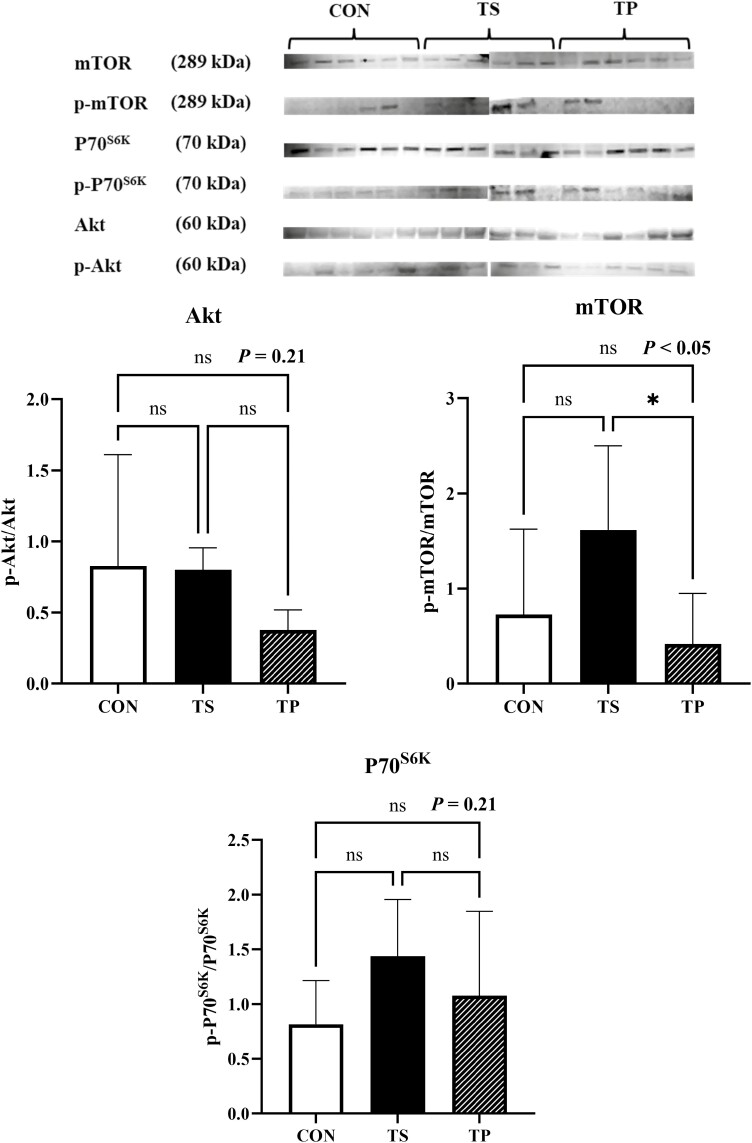
The protein levels of Akt, mTOR, and P70^S6K^ in LD muscle of beef steers under temperature swing and transport conditions. Error bars indicate the standard error. The *P*-values are determined by one-way ANOVA (*P < 0.05, **P < 0.01).

The stressors employed in this study did not induce any significant changes in the Akt/mTOR/P70^S6K^ pathway (Figure 5). Specifically, the phosphorylation of Akt was unaffected (*P* = 0.21) by the treatment. However, the ratio of phosphorylated mTOR to total mTOR was found to be higher (*P* < 0.05) in the TP group compared to the CON group but did not differ significantly from the CON group. In addition, the treatments did not significantly affect the protein abundance of P70S6K (*P* = 0.21).

### Adipogenic genes

**Figure 6.**
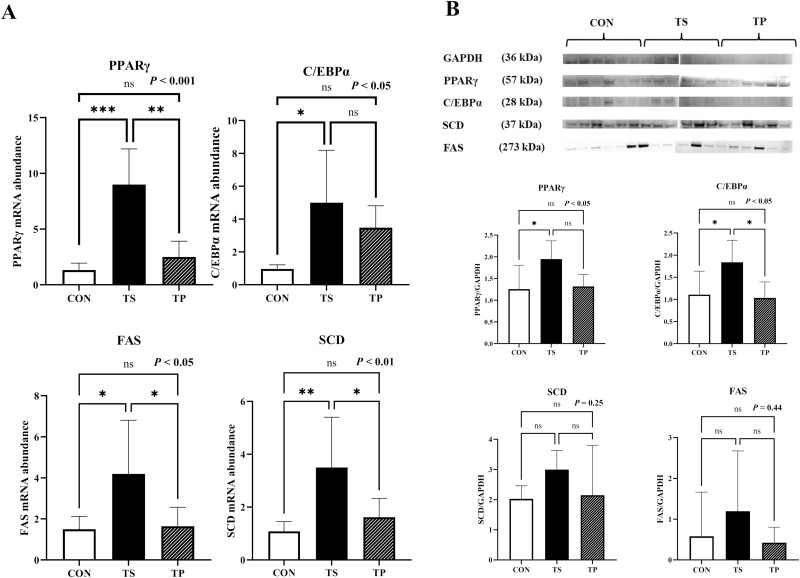
mRNA expression and protein abundance levels of adipogenesis markers (PPARγ, C/EBPα, FAS, and SCD) in beef steers under temperature swing and transport conditions. (**A**) *PPARγ*, *C/EBPα*, *FAS*, and *SCD* mRNA levels in CON, TS, and TP groups. (**B**) Western blots (top) and quantification of protein levels (bottom) of PPARγ, C/EBPα, SCD, and FAS in CON, TS, and TP groups. Error bars indicate the standard error. The *P*-values are determined by one-way ANOVA (*P < 0.05, **P < 0.01).

Steers subjected to the TS treatment exhibited a significant increase (*P* < 0.01) in the mRNA gene expression of *PPARγ* compared to the CON and TP groups (Figure 6A). This finding was further confirmed by protein analysis, which revealed that the TS treatment significantly increased (*P* < 0.05) the protein abundance of PPARγ (Figure 6B). In addition, *C/EBPα* expression was upregulated (*P* < 0.05) by the TS treatment compared to the CON group (Figure 6A). Similar to the mRNA expression, the protein level analysis demonstrated that the TS treatment increased (*P* < 0.05) the protein level of C/EBPα compared to the CON group (Figure 6B). Furthermore, the expression of *FAS* was greater (*P* < 0.05) in muscle samples from TS steers compared to the CON and TP groups (Figure 6A), although no change (*P* = 0.44) was detected in the protein level (Figure 6B). The TS steers also exhibited higher (*P* < 0.01) expression of *SCD* compared to the CON and TP groups (Figure 6A), although no change (*P* = 0.25) was detected in the protein level (Figure 6B).

## Discussion

Severe environmental changes elicit various cellular and immune responses in animals to uphold body homeostasis. Elevated ambient temperatures expose animals to heightened core temperature, thereby raising muscle temperature. Existing research demonstrates that heat exposure induces notable alterations in cellular processes within skeletal muscle, particularly affecting satellite cells responsible for muscle regeneration and hypertrophy, while the results vary. For instance, broiler chickens subjected to a temperature of 32 °C for a duration of 14 days exhibited reduced yield in breast muscle due to the downregulation of key factors such as IGF-1, IGF-1 receptor, mTOR, and myogenin expression (Ma et al., 2018). In contrast, HS has been shown to enhance the proliferation and differentiation of satellite cells in both humans and turkeys (Clark et al., 2016; Xu et al., 2022).

A scarcity of studies has explored the effects of temperature swings, as opposed to prolonged exposure to constant high-HS, particularly in beef cattle. It is hypothesized that cattle residing in regions characterized by alternating cold and hot days are more likely to experience significant temperature fluctuations rather than enduring continuous high-heat conditions.

In our present investigation, the steers in the TS group experienced a temperature fluctuation exceeding 20 °C along with a THI change of more than 30 units for a duration of 3 h. These environmental conditions elicited significant behavioral responses indicating HS in the steers. The observed responses included extended tongue protrusion, reduced appetite, excessive salivation, and a notable increase in respiratory rate. The normal respiration rate for cattle is considered to be approximately 26 to 50 breaths per minute, with breaths exceeding 120 deemed to be severe HS classification (Becker et al., 2020). All of the behavioral signs exhibited by the TS steers indicate extreme HS. Notably, the THI to which the steers were exposed slightly exceeded the comfort threshold, with a value greater than 72 (Polsky and von Keyserlingk, 2017). This provides external evidence indicating that significant temperature fluctuations can induce thermal stress in beef cattle, even if the overall temperature level is not excessively high.

On the other hand, the rectal temperatures of the TS group were observed to be slightly lower compared to the CON group. However, this discrepancy appears to be attributed to individual variations among the animals rather than an effect directly related to the TS treatment. Rectal temperature measurement is commonly employed as a noninvasive method for detecting heat in cows or assessing HS. Nevertheless, it is important to note that the accuracy of rectal temperature measurements can be influenced by the specific procedure employed, such as the depth of insertion of the thermometer into the rectum and the potential presence of feces (Burfeind et al., 2010). It is also plausible that the rapid alteration in core temperature may not accurately reflect the rectal temperature. In our study, the cattle were exposed to the TS conditions for a period of 3 h, which might not have been sufficient time to observe significant changes in rectal temperature. Bottcher and Hoff (1997) demonstrated that body temperature responses to thermal stressors were delayed, occurring between 3 and 5 h postexposure. This could account for the absence of observed changes in rectal temperature, as the timing fell outside the measurement sampling window. Additionally, the lack of correlation between cellular responses and alterations in core body temperature may be due to the fact that a THI of 72 is not sufficiently extreme to disrupt thermoregulatory homeostasis. Further investigation is warranted to elucidate the underlying mechanisms.

Furthermore, it was observed that the serum cortisol level remained unaffected by the temperature treatments. This aligns with previous discussions on the limitations of serum cortisol as a stress measurement in animals. Fluctuations in cortisol levels can occur rapidly in response to different stressors, and factors such as time, individual variability, and sample collection and handling can introduce variability in cortisol measurements (Moberg, 2000). Steers in the trial underwent movement through the chute and were temporarily removed from their pen for blood collection, which could have potentially elevated the baseline level of serum cortisol.

The physiological response to thermal stress is intertwined with the role of HSPs as early responders in situations of stress, such as changes in temperature, physical exertion, or disease (Lee et al., 2007). HSPs function as molecular chaperones, regulating various molecular signals to prevent cellular damage (Collier et al., 2008; Becker et al., 2020; Kim et al., 2020a). In skeletal muscle, HSPs are known to contribute to the synthesis of new proteins, the stabilization of native protein structure, the reduction of damage, and the repair of damaged proteins (Oishi et al., 2009). Limited data are available, particularly in beef cattle, regarding the response of HSPs when animals are exposed to severe fluctuations in temperature. Reith et al. (2022) conducted a transcriptomic analysis of skeletal muscle biopsies from beef steers exposed to a THI of 81. They observed upregulation of NUP54, LOC101903734, HIF1A, DCBLD2, HNRNPU, and PDZD9 genes; however, no changes in HSPs expression were detected. In our current study, the steers in the TS group demonstrated pronounced overexpression of HSPs at both the gene and protein levels. In a previous in vitro model utilizing bovine satellite cells, we found that HSP isoforms reached their peak expression 3 h after exposure to heat (41 °C), returning to baseline levels after 6 h, except for HSP27 (Kim et al., 2023). Furthermore, the expression of HSP70 was shown to be highly responsive to extreme heat exposure, exhibiting an 80-fold increase compared to the thermal-neutral group. HSP70 is widely ­recognized as the most responsive HSP to HS and exhibits a strong correlation with ambient HS in cattle (Gaughan et al., 2013; Kim et al., 2020b). Nevertheless, our data revealed that TS did not influence the expression or activity of HSP 70. This observation implies that the functional characteristics of HSPs in response to varying temperature fluctuations may diverge from their responses under constant high-heat conditions.

Transportation is acknowledged as a notable detrimental stressor involving physical and psychological factors experienced by cattle, which can potentially impact both animal growth and meat quality (Swanson and Morrow-Tesch, 2001). Hu et al. (2020) also reported that a 2-h transportation period led to increased mRNA levels of HSP27 and HSP70 in the liver and lungs of goats. However, our research findings demonstrate that the upregulation of HSPs was significantly more pronounced in response to severe temperature fluctuations compared to transportation stress. This suggests that the magnitude of stress experienced by cattle under temperature fluctuations surpasses that induced during transportation.

Concomitant with HSPs, TS appeared to modulate the signaling pathways involving early regulators of skeletal muscle growth. Specifically, we observed an augmentation in the expression of Pax7 and Myf5 in steers exposed to TS, while MyoD and MyoG remained unaffected. HS induces skeletal muscle damage, leading to the activation of satellite cells for muscle regeneration (Chal and Pourquié, 2017). It has been reported that HS-induced satellite cell proliferation in turkey (Clark et al., 2016; Xu et al., 2021) and swine (Kamanga-Sollo et al., 2011). Previous studies have reported alterations in the expression of Pax7 and other MRFs due to HS. For instance, Park et al. (2023) reported that exposure to 41 °C heat for 5 days downregulated Pax7 but increased MyoD and MyoG in Porcine Muscle Satellite Cells (PMSCs). Our recent study involving bovine myocytes reveals a similar trend: short-term heat exposure led to the upregulation of myogenic regulatory factors (Kim and Kim, 2023).

A similar scenario may occur in skeletal muscles under conditions of severe temperature fluctuations. Human ­studies have shown that rapid changes in skin temperature can induce muscle damage (Da Silva et al., 2018). However, there is limited research available on how temperature fluctuations regulate MRFs and ultimately impact skeletal muscle hypertrophy and regeneration. Pax7 and Myf5 are genes expressed during the early stages of myogenic differentiation in satellite cells and skeletal muscle regeneration. Understanding the mechanisms by which temperature variations influence these MRFs is crucial for elucidating the broader implications on skeletal muscle health and recovery.

Our data further revealed that the three primary isoforms of MHC in bovine skeletal muscle, namely *MHC I*, *IIA*, and *IIX*, were all increased in response to TS. Myosin heavy chains (MHCs) are crucial contractile proteins, and their isoforms serve as sarcomeric markers for skeletal muscle (Agbulut et al., 2003). A previous study has also reported that the simultaneous knockdown of MHCs induced skeletal muscle hypoplasia (Hitachi et al., 2023). The observed increase in MHC expression suggests an augmented demand for contractile properties and postnatal muscle hyperplasia during temperature challenges.

Previous research has demonstrated the activation of the IGF-1/Akt/mTOR pathway in response to temperature elevation in various species, including rodents (Yoshihara et al., 2013), turkey (Xu et al., 2022), and humans (Kakigi et al., 2011). The IGF-1/Akt/mTOR pathway is well-known for its role in promoting protein synthesis in skeletal muscle, thus suggesting an anabolic effect of heat exposure. In our study, we observed an increase in mRNA expression of IGF-1 in the TS group, while the phosphorylation of Akt, mTOR, and P70^S6K^ remained unchanged. The observed elevation in IGF-1 mRNA expression in the muscle biopsy suggests the potential activation of the IGF-1-mediated mTOR pathway in subsequent stages. However, it is essential to note that our investigation was limited to a 3-h exposure to TS, which might be insufficient to detect the entire downstream cascade of events. Further studies with longer durations of TS exposure are warranted to comprehensively elucidate the subsequent signaling processes and their implications.

Our data present compelling empirical evidence demonstrating the upregulation of crucial transcription factors involved in the differentiation of adipocytes and the mechanisms governing lipid accumulation due to TS. These transcription factors include PPARγ and C/EBPα, which are known as primary regulators of adipogenesis, as well as FAS and SCD. The analysis was performed on biopsied samples obtained from the *longissimus muscle* area. Therefore, the observed outcomes might be linked to the growth of adipose tissue within or between muscle fibers. This molecular modification observed in the longissimus muscle exhibited similar patterns to those seen during extreme heat exposure in animals. For instance, prior studies have demonstrated that HS can enhance the potential for adipose tissue accumulation by activating PPARγ. Various investigations have been conducted on different livestock species, such as swine (Jiang et al., 2007; Lu et al., 2014; Huang et al., 2021), dairy cows (Kra et al., 2021), bulls (Chen et al., 2022), which have consistently reported an upregulation of PPARγ expression under HS conditions. Another crucial transcription factor involved in adipocyte differentiation, C/EBPα, exhibited an increase after a 3-h period of thermal stress. Similar trends have been observed in studies examining HS induction on transcriptomes associated with adipogenesis. Huang et al. (2021) also reported a significant increase in PPARγ and C/EBPα expression following exposure to 41.5 °C in 3T3-L1 preadipocytes. Thermal variations, particularly HS, have been observed to induce alterations in lipid metabolism in livestock animals. It has been proposed that HS can modify adipose tissue through transcriptional or hormonal mechanisms. In a rodent model, a 6-h period of HS demonstrated a tendency to increase the expression of the PPAR-γ co-activator-1α (PGC-1α) gene in the soleus and tibialis anterior muscles (Sanders et al., 2009). In a swine study, HS resulted in increased expression of PCK1 and AP2 in subcutaneous fat, along with elevated HSP70 expression (Qu, 2018). In another swine study, Kouba et al. (2001) reported increased back fat and lipoprotein lipase (LPL) activity in back fat and flared fat adipose tissue. In contrast, elevated temperatures were found to have an opposing effect, reducing the proportion of intramuscular fat and suppressing the process of de novo synthesis of fatty acids in pigs. This decrease was attributed to the inhibition of acetyl coenzyme A carboxylase activity. Similarly, chronic exposure to heat was observed to inhibit the activities of lipolytic enzymes and decrease non-esterified fatty acid (NEFA) levels in broilers (Geraert et al., 1996). The regulatory mechanisms by which thermal changes influence adipose tissue expansion, metabolism, and the subsequent impact on final products remain unclear. Additional screening studies, particularly in the bovine species, are needed to gain further insights into these processes.

## Conclusion

In conclusion, severe environmental changes, such as elevated ambient temperatures and temperature fluctuations, can elicit cellular and immune responses in animals, ­including ­skeletal muscle and adipose tissue. Temperature fluctuations can induce responses similar to HS in beef cattle, even without excessively high overall temperatures, as evidenced by behavioral signs and increased expression of HSPs. The response of HSPs to temperature fluctuations may differ from that under sustained high-heat conditions. Temperature swings also affect myogenic regulatory factors, myosin heavy chain expression, and the IGF-1/Akt/Mtor pathway, potentially influencing skeletal muscle growth. Furthermore, temperature swings can modulate the differentiation of adipocytes and mechanisms governing lipid accumulation, involving transcription factors such as PPARγ and C/EBPα. However, the long-term effects of these changes and the underlying regulatory mechanisms require further investigation, particularly in bovine species.

## Abbreviations:

AT: ambient temperature
BW: body weight
DMI: dry matter intake
DDGS: distiller’s dried grains with soluble
HMC: high-moisture corn
HS: heat stress
LM: Longissimus muscle
mRNA: messenger ribonucleic acid
TNZ: thermal neutral zone
PMSC: placental mesenchymal stromal cells
RR: respiratory rate
RT: rectal temperature
HSPs: heat shock proteins
THI: Temperature-Humidity Index
Pax7: paired box gene 7
Myf5: myogenic factor 5
MHC: myosin heavy chain
PPARγ: peroxisome proliferator-activated receptor gamma
C/EBPα: CCAAT/enhancer-binding protein-alpha
FAS: fatty acid synthase
SCD: stearoyl-coenzyme A desaturase
